# Pristane-Accelerated Autoimmune Disease in (SWR X NZB) F1 Mice Leads to Prominent Tubulointerstitial Inflammation and Human Lupus Nephritis-Like Fibrosis

**DOI:** 10.1371/journal.pone.0164423

**Published:** 2016-10-19

**Authors:** Agnes Gardet, Wei C. Chou, Taylor L. Reynolds, Diana B. Velez, Kai Fu, Julia M. Czerkowicz, Jeffrey Bajko, Ann M. Ranger, Normand Allaire, Hannah M. Kerns, Sarah Ryan, Holly M. Legault, Robert W. Dunstan, Robert Lafyatis, Matvey Lukashev, Joanne L. Viney, Jeffrey L. Browning, Dania Rabah

**Affiliations:** 1 Biogen, Cambridge, Massachusetts, United States of America; 2 Division of Rheumatology and Clinical Immunology, University of Pittsburgh, Pittsburgh, United States of America; 3 Boston University School of Medicine, Department of Microbiology, Boston, United States of America; Universite Paris-Sud, FRANCE

## Abstract

Mouse models lupus nephritis (LN) have provided important insights into disease pathogenesis, although none have been able to recapitulate all features of the human disease. Using comprehensive longitudinal analyses, we characterized a novel accelerated mouse model of lupus using pristane treatment in SNF1 (SWR X NZB F1) lupus prone mice (pristane-SNF1 mice). Pristane treatment in SNF1 mice accelerated the onset and progression of proteinuria, autoantibody production, immune complex deposition and development of renal lesions. At week 14, the pristane-SNF1 model recapitulated kidney disease parameters and molecular signatures seen in spontaneous disease in 36 week-old SNF1 mice and in a traditional IFNα-accelerated NZB X NZW F1 (BWF1) model. Blood transcriptome analysis revealed interferon, plasma cell, neutrophil, T-cell and protein synthesis signatures in the pristane-SNF1 model, all known to be present in the human disease. The pristane-SNF1 model appears to be particularly useful for preclinical research, robustly exhibiting many characteristics reminiscent of human disease. These include i) a stronger upregulation of the cytosolic nucleic acid sensing pathway, which is thought to be key component of the pathogenesis of the human disease, and ii) more prominent kidney interstitial inflammation and fibrosis, which have been both associated with poor prognosis in human LN. To our knowledge, this is the only accelerated model of LN that exhibits a robust tubulointerstitial inflammatory and fibrosis response. Taken together our data show that the pristane-SNF1 model is a novel accelerated model of LN with key features similar to human disease.

## Introduction

Lupus nephritis (LN) is a heterogeneous disease that presents with a broad spectrum of clinical and pathologic manifestations. Although immune complex mediated glomerulonephritis is the most common type of renal disease, tubulointerstitial inflammation and fibrosis are also important components of LN [1,2]. Several spontaneous murine models of LN exist, including BWF1 (NZB X NZW F1), SNF1 (SWR X NZB F1), MRL/lpr, in addition to congenic mouse models and many strains of gene targeted mice that present with features of LN [3]. Lupus presents many challenges for preclinical assessment of new therapeutic candidates. In addition to the multitude of pathogenic mechanisms that could impact disease development, the significant time period required to develop disease in SLE prone mice poses an important hurdle to the pre-clinical modeling of lupus. Considerable effort has been devoted to establish accelerated mouse models of SLE that are relevant to human disease.

Type I interferon (IFN-I) has been suggested to play a key role in SLE pathogenesis and IFN delivered exogenously with an adenovirus encoding IFNα (Adv-IFNα) or stimulated by poly (I:C), has been used to accelerate disease in lupus prone mice [4–7]. Adv-IFNα delivery in BWF1 (Adv-IFNα BWF1) is a well-established and widely used IFN-I accelerated mouse model. While this model recapitulates immune complex glomerulonephritis it is not a robust model for tubulointerstitial inflammation [8].

Tetramethylpentadecane, also known as pristane, is hydrocarbon oil known to induce lupus like disease in non-autoimmune mice strains and accelerate disease in lupus-prone BWF1 mice [9,10]. Other oil adjuvants such as (Incomplete Freund's adjuvant, IFA) and squalene (MF59) have been shown to induce lupus-related autoantibodies in non-autoimmune mice [11]. Recently, IFA have been shown to accelerate the onset and progression of proteinuria in BWF1 mice [12].

Pristane-mediated disease is characterized by hypergammaglobulinemia, autoantibody production and immune complex glomerulonephritis. Pristane induced LN is another model where IFN-I has been shown to be central to disease development [9]. IFN-I production in pristane-treated animals appears to be TLR7 and IRF5 dependent and IFN-I signaling through IFNAR is essential for the development of autoimmunity in pristane-induced disease [13,14].

In this study, we describe a comprehensive longitudinal characterization of a novel model that overlays pristane on the genetic lupus prone background of SNF1 mice and provide a valuable tool for modeling human disease. In contrast to BWF1 mice, pristane injection in SNF1 mice led to a more rapid disease with proteinuria and immune-complex deposition detected as early as 5 weeks post-pristane. The pristane-SNF1 model appears to accentuate pathologies that differ from other accelerated models such as the Adv-IFNα BWF1 model. Importantly, the pristane-SNF1 model exhibited several characteristics that robustly replicate what is seen in human disease, including the activation of cytosolic DNA sensing mechanisms and prominent inflammation and fibrosis. Thus, the pristane-SNF1 model may be a valuable tool for preclinical assessment of new therapeutic candidates.

## Material and Methods

### Mice

SNF1 females were bred and housed at Biogen in a standard ABL1 room. BWF1 were obtained from Jackson Laboratories (Bar Harbor, ME). All animals were maintained at the animal facility of Biogen in controlled temperature rooms, with light-dark cycles, and with free access to food and water. Standard environment enrichment of nestles was provided. All animal procedures were conducted in accordance with Biogen guidelines and applicable animal research regulations and were approved by the Institutional Animal Care and Use Committees at Biogen.

### Assessment of disease

Starting at 8 weeks of age, animals were weighed and albuminuria was measured weekly. Daily observations included monitoring animals for physical condition and for clinical signs of disease which included rough coat, edema, labored breathing and respiratory distress. In order to alleviate suffering, moribund animals were euthanized by CO2 inhalation after two consecutive readings of ≥ 1000 mg/dl of albuminuria and/or when they exhibited respiratory distress. Using these criteria animals were euthanized before they became severely ill. On two occasions, an animal was found dead without having clinical signs of disease or elevated albuminuria. Kidney disease was evaluated using a number of serum and urine markers of glomerular/tubular leakage and kidney function. Urine was analyzed for total protein which was used as a primary end point. Serum was analyzed for blood urea nitrogen (BUN), creatinine, and cholesterol. All measurements were done using the Beckman Coulter AU400.

### LN disease model

Pristane-SNF1 model: 9 week-old female SNF1 mice were injected intraperitoneally (i.p.) with 500μl of pristane (2, 6, 10, 14-tetramethylpentadecane, Sigma). Age-matched SNF1 mice were used as controls.

Spontaneous SNF1 model: 36 week-old female SNF1 mice with proteinuria greater than 1000 mg/dl were compared to pristane-SNF1 mice.

Adv-IFNα BWF1 model: 9 week-old female BWF1 mice were injected with an adenovirus expression vector containing recombinant mouse IFNα gene cassette Adv-IFNα. Mice were injected intravenously (i.v.) with 5 X 10^9^ viral particles [4].

Blood, serum, urine, kidney and spleen were collected at weekly intervals for various endpoint analyses to assess disease progression. Two independent longitudinal experiments were conducted with pristane-SNF1 mice, Adv-IFNα BWF1 mice and age-matched controls.

### Efficacy study

Mice received i.p. injection of either hamster anti-CD40L (Biogen) or its isotype control (anti-Ha 4/8) or PBS. Anti-CD40L antibody and its isotype control were administered 3 weeks post-pristane and treated every 3 days until the experiment was terminated. Proteinuria was measured weekly starting at week 7 through week 12 post-pristane. All mice were euthanized 14 weeks post-pristane for various endpoint analyses.

### Microarray data collection

Snap frozen whole blood and kidneys were homogenized in Trizol reagent (Invitrogen corporation) and purified according to the manufacturer’s protocol. Total RNA was further purified using an RNeasy mini column (Qiagen Sciences) and treated with DNase I amplification grade (Invitrogen).

Sample labeling and hybridization were performed using HT-MG-430 PM plates (Affymetrix Inc)

### Microarray data analysis

All microarray mouse transcript profiling data are deposited in the Gene Expression Omnibus (GEO) database (GSE86423, GSE86424 and GSE86425). Public microarray with human LN kidneys was obtained from GEO database (GSE37460). Mouse microarray datasets were analyzed using R software (version 2.11.1; R Foundation, Vienna, Austria) and Bioconductor computational tools. Probe intensity values were normalized using Robust Multi-array Average (RMA) normalization. The differential expression analysis was done using the Linear Models for Microarray (LIMMA) data analysis [15]. Human microarray data were analyzed using Partek® Genomics Suite® software (version 6.6 Copyright 2014). After RMA normalization, the differential expression analysis was done using ANOVA. All p-values for each probe were adjusted using False Discovery Rate (FDR) using the Benjamini and Hochberg method [16]. Statistical significance was defined as FDR adjusted p-value < 0.05. Z-scores and heatmaps were generated using Gene-E software (Broad Institute, Boston, MA).

### Measurement of antibodies

Serum ANA and anti-dsDNA antibodies (Total IgM, IgG, IgA) were measured using kits from Alpha Diagnostics. Clot-free sera were diluted up to 1:1000 for autoantibody analysis. Serum IgG isotypes were measured using Luminex multiplex bead array from Millipore.

### Histologic analysis of kidneys

Formalin-fixed kidneys were processed, embedded in paraffin, and replicate sections were stained with H&E, periodic acid Schiff (PAS), and Jone’s Basement Membrane stains. These slides were scored blind using a semi-quantitative scoring system from 0–3 for glomerulopathy, tubular dilation and infiltrates (0: no change, 1: minimal, 2: mild, 3: marked) and from 0–4 for fibrosis (1: minimal, 2: mild, 3: moderate and 4: marked).

### Immunohistochemistry and RNAscope

For detection of immune complexes, frozen kidney sections were fixed with acetone and stained with a mixture of FITC-conjugated rat anti-mouse IgG1, IgG2a/2b, IgG3 (BD Pharmingen), IgG2c (Southern Biotech) A mixture of FITC-conjugated rat isotypes IgG1, IgG2a, and IgG2b were used as controls (BD Pharmingen).

For detection of fibrosis markers, 5 μm sections from formalin-fixed kidneys of mice with matched levels of proteinuria were stained with Masson’s Trichrome stain, or anti-TNC antibody (R&D System, clone 578) and anti-ASMA (Abcam, clone 1A4) with Ventana automated staining system (Ventana Medical Systems, Tuscon, AZ, USA). Needle biopsies from 5 patients with LN (Class II, 1 patient; Class III, 1 patient; Class IV, 1 patient; Class IV/V, 2 patients) were obtained from Folio Biosciences (Powell, OH) and stained using the same methods.

For *Aim2*, *Sting*, *Ifih1* mRNA detection, 5μm sections of formalin-fixed mouse kidneys with matched levels of proteinuria were probed with antisense RNAscope oligomers (Advanced Cell Diagnostics, Hayward, CA) and detected using Ventana automated staining system (Ventana Medical Systems, Tuscon, AZ, USA). Bacterial DapB probe was used as negative control. Stained area was quantified from cortex (Two 400X600 μm fields per animal) using Image J software [17,18] (S1 Fig). Statistical significance was assessed using unpaired two-tailed Student’s t-test.

## Results

### Pristane treatment accelerates disease in SNF1 mice

**Fig 1:**
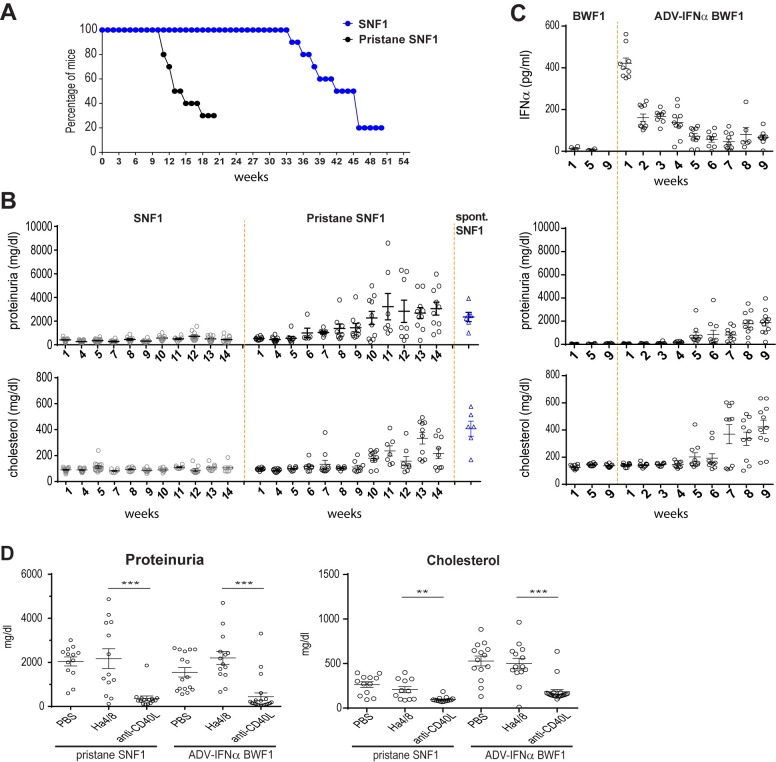
Pristane treatment accelerates disease in SNF1 lupus-prone mice. (A) Percentage of non-moribond mice in weeks after birth (SNF1 n = 15) or in weeks after pristane injection (pristane-SNF1 n = 15). Shown is a representative plot of 2 independent experiments. (B) Progression of proteinuria in pristane-treated, untreated age matched controls, and 36-week old SNF1 mice with spontaneous disease. Each symbol indicates an individual mouse. (C) Serum IFNα concentration, and progression of proteinuria and serum cholesterol elevation in Adv-IFNα BWF1 model and untreated age matched controls. Each symbol indicates an individual mouse. (D) Effect of CD40L and BAFF blockade on proteinuria and serum cholesterol in pristane-SNF1 mice (14 weeks after treatment) or Adv-IFNα BWF1 (9 weeks after treatment) either treated with anti-CD40L (MR1), or the isotype control (Ha 4/8) and compared with PBS treated mice. Each symbol indicates an individual mouse. This is a representative experiment of 2 independent experiments. Statistical significance was assessed using One-way Anova test.

Pristane was injected in 9-week old SNF1 mice, prior to clinical signs of spontaneous disease. Morbidity was observed at 12–14 weeks post-pristane (Fig 1A) and coincided with high BUN and serum creatinine reflecting renal failure (data not shown). Pristane greatly accelerated the disease onset in the SNF1 strain with proteinuria becoming evident at 7–8 weeks post pristane injection (Fig 1B). All the SNF1 mice were severely proteinuric (>1000 mg/dl) at 14 weeks post pristane injection with levels similar to 36 week-old SNF1 mice with spontaneous disease (Fig 1B). Disease acceleration upon pristane treatment was more dramatic in the SNF1 strain compared to BWF1 strain. In the SNF1 mice, onset of proteinuria was observed at 7 weeks post- pristane injection, while it occurred at 13 weeks after pristane injection in the BWF1 strain as previously reported [10] (S2 Fig).

The progression of disease in pristane-SNF1 model was compared to another established accelerated mouse model of lupus nephritis, IFNα-accelerated BWF1 model (Adv-IFNα BWF1) [4]. Interferon was delivered using an adenovirus vector to 8 week-old BWF1 mice leading to a sharp rise in IFNα one week post-adenoviral delivery and declining to a steady state at 3 weeks post-treatment (Fig 1C). Onset of proteinuria was detected at 5 weeks post IFNα delivery (Fig 1C). Morbidity due to renal failure (high BUN and serum creatinine) was observed 7–9 weeks post-ADV-IFNα injection (data not shown).

To ensure that pristane treatment of SNF1 mice did not lead to an aggressive pathology that can no longer respond to therapeutic interventions, we tested whether CD40L blockade could restore kidney function in this model as it was described efficacious in the spontaneous SNF1 model [19,20]. Three weeks after pristane administration SNF1 mice were treated with 10 mg/kg anti-CD40L (MR1) twice a week for 9 weeks. Administration of anti-CD40L to pristane-SNF1 mice significantly inhibited proteinuria similar to the efficacy seen in Adv-IFNα BWF1 model (Fig 1D) highlighting that the disease development in the pristane-SNF1 model can be inhibited by therapeutic interventions.

Together, these results show that the pristane-SNF1 model is an accelerated mouse model of lupus that recapitulates some of the clinical features and pathogenic mechanisms observed in both spontaneous and accelerated mouse models of SLE as well as human SLE.

### Pristane treatment leads to hypergammaglobulinemia, autoantibody production and early deposition of immune complexes in the kidneys of SNF1 mice

**Fig 2:**
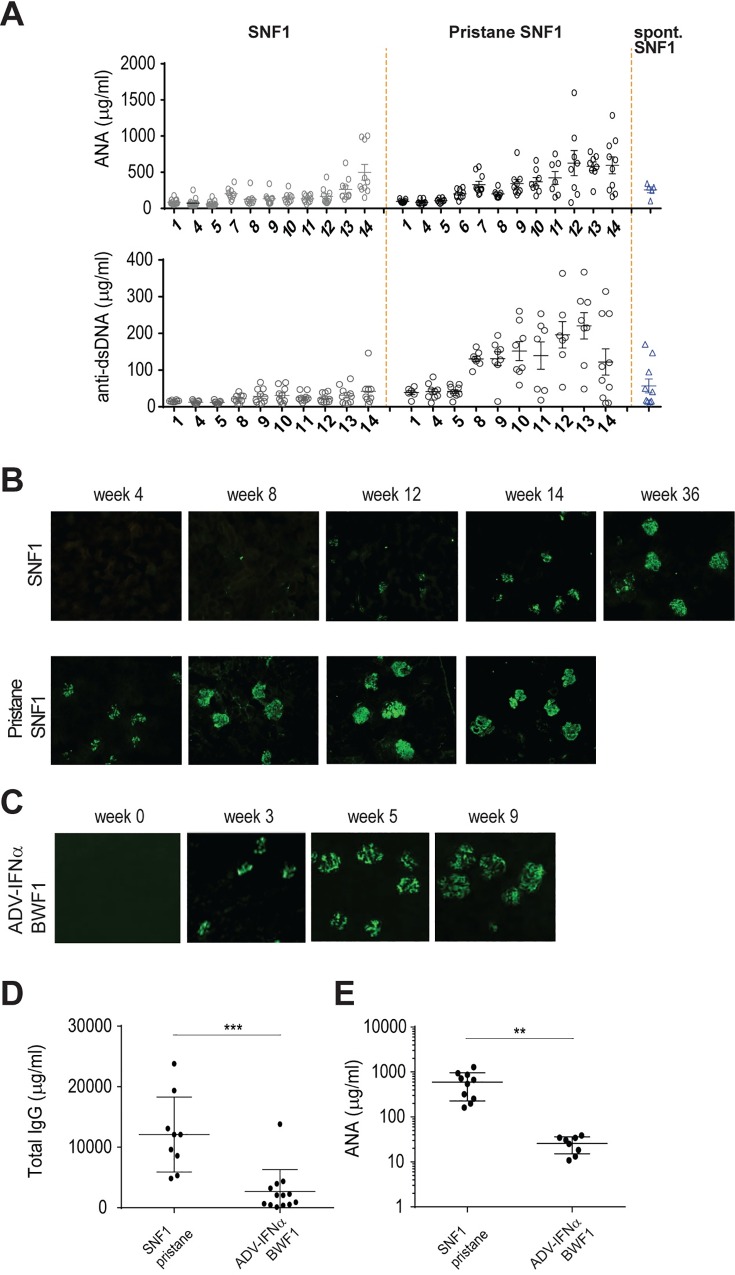
Serum antibody levels and kidney immune complex deposition in the pristane-SNF1 model and in the Adv-IFNα BWF1 model. (A) Titers of serum anti-nucleic acid antibodies (ANA) and anti-dsDNA antibodies (Total IgM, IgA, IgG in μg equivalents/ml) in pristane-treated, untreated age matched controls, and 36-week old SNF1 mice with spontaneous disease. (B) Frozen kidney sections from pristane-treated SNF1 mice on weeks 4, 8, 12, and 14, age match control and 36-week old SNF1 mice with spontaneous disease were stained with FITC conjugated anti-IgG Ab to detect immune complex deposition. Staining is representative of 4 mice examined in each group. (C) Frozen kidney sections from Adv-IFNα BWF1 mice on weeks 0, 3, 5, and 9 were stained with FITC conjugated anti-IgG Ab to detect immune complex deposition. Staining is representative of 4 mice examined in each group. (D) Titers of serum total IgG antibodies, anti-nucleic acid antibodies (ANA) were compared between diseased SNF1 pristane mice (14 weeks after treatment) and Adv-IFNα BWF1 mice (9 weeks after treatment). Statistical significance was assessed using unpaired two-tailed Student’s t-test (*** p<0.001, **p<0.01).

Hallmarks of LN include the presence of autoantibodies and immune complex (IC) deposition in the kidney. In pristane-SNF1 mice, serum ANA and anti-dsDNA autoantibodies increased at 7–8 week post pristane treatment (Fig 2A), which coincided with the onset of proteinuria (Fig 1B). Autoantibody levels were significantly higher in pristane-SNF1 mice than in the 36 week old SNF1 spontaneous model (Fig 2A). Kidney immune complex deposition was detected at week 4 after pristane injection in the pristane-SNF1 model and at week 3 in the ADV-IFNα BWF1 model (Fig 2B and Fig 2C). While both models showed accelerated production of ANA and IC deposition, pristane-SNF1 led to a more significant increase in total IgG and autoantibody levels compared with ADV-IFNα BWF1 (4 fold and 14 fold difference respectively) (Fig 2D and Fig 2E).

### Pristane treatment induces kidney glomerulonephritis, tubular dilatation and fibrosis in SNF1 mice

**Fig 3:**
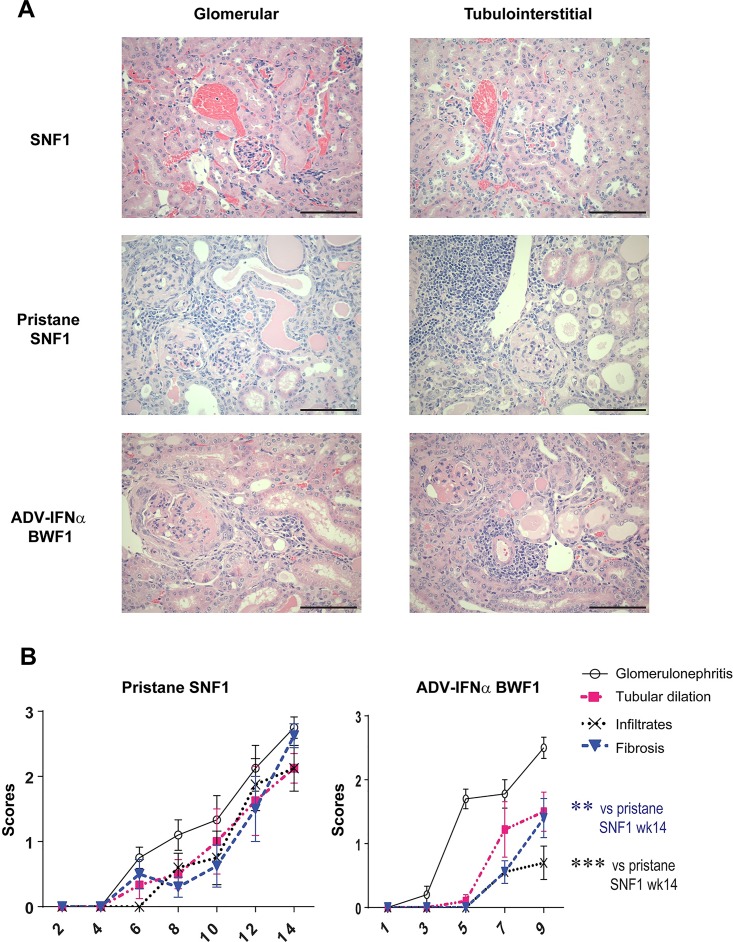
Kidney morphology in pristane-SNF1 model and Adv-IFNα BWF1 model. (*A)* Glomerular and tubulointerstitial changes in diseased pristane-SNF1 (14 weeks after treatment) and Adv-IFNα-BWF1 models (9 week after treatment) observed using H&E stained kidneys sections. Animals were matched for proteinuria (B) H&E sections of kidneys collected on the weeks indicated from pristane-treated SNF1 and Adv-IFNα injected BWF1 mice were scored for glomerulopathy, tubular dilation, fibrosis and inflammatory infiltrates. The lines represent the average scores, vertical lines represent SEM. Statistical significance was assessed using unpaired two-tailed Student’s t-test (*** p<0.001, **p<0.01). At least 8 animals were analyzed for scoring for each group, except pristane-SNF1 groups at week 4 and week 6 (5 animals per group).

Kidney sections from pristane-SNF1 mice, Adv-IFNα BWF1 mice, and spontaneous disease controls (SNF1 and BWF1 mice) were blindly scored for glomerulopathy, tubular injury, interstitial inflammation and fibrosis using H&E stained tissue sections. The renal lesions in pristane-SNF1 mice were qualitatively similar to those that developed spontaneously at later time points in SNF1 and BWF1 mice (data not shown) and to those induced by Adv-IFNα in BWF1 (Fig 3A). Pristane-SNF1 mice uniformly exhibited moderate to severe diffuse proliferative glomerulonephritis 12–14 weeks after pristane injection. Glomerular changes in proteinuric mice revealed basement membrane thickening, mesangial cell proliferation, hyper-segmentation and sclerosis of glomerular tufts, and sclerosis of Bowman’s capsule (Fig 3A). Glomerular changes were accompanied by tubular injury ranging from cytoplasmic basophilia to overall atrophy and dilation. Interstitial inflammation was pronounced and characterized by abundant interstitial peripelvic aggregates lining tubules and surrounding glomeruli. Interstitial fibrosis intersected tubules and often surrounded degenerative tubular areas and/or affected glomeruli (Fig 3A).

The pristane-SNF1 mice displayed higher levels of tubulointerstitial inflammation with large immune infiltrates and fibrosis compared to Adv-IFNα BWF1 mice (Fig 3A and Fig 3B). In addition, tubulointerstitial damage, which is generally a feature of end-stage renal disease, developed relatively early in the disease course of the pristane-SNF1 model (Fig 3B). Therefore, pristane injection in SNF1 mice not only accelerated nephritis but also induced more interstitial lesions and fibrosis than which have been both associated with poor prognosis in human LN [21–24].

### Transcriptional changes in kidneys in the pristane-SNF1 bear hallmarks of human lupus nephritis

To further characterize the kidney disease in pristane-SNF1 mice, we analyzed gene expression of kidney tissues obtained from pristane- SNF1 mice, spontaneous 36 week-old SNF1 mice Adv-IFNα BWF1 mice and untreated matched controls. We detected 952 differentially expressed genes in kidneys from pristane SNF1 mice and 741 differentially expressed genes from Adv-IFNα BWF1 mice compared to age-matched controls (> 2-fold compared to age-matched controls, FDR adjusted p<0.05) (S1 File).

**Fig 4:**
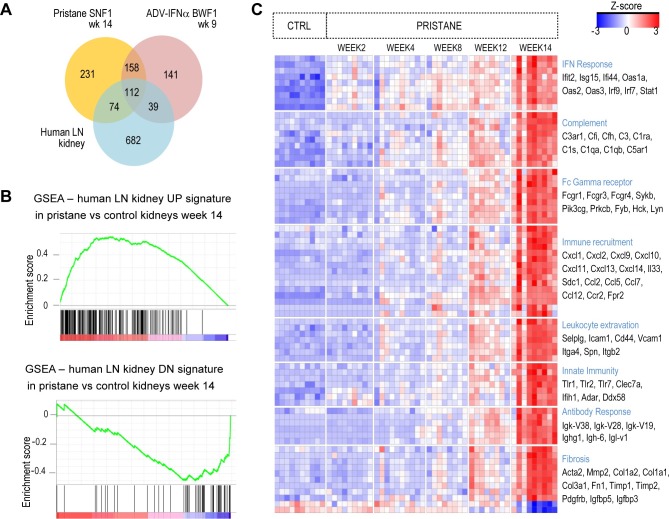
Kidney transcriptional response of pristane-SNF1 model and Adv-IFNα BWF1. (A) Differentially expressed genes were identified using LIMMA package for both mouse models (with fold change difference > |2| and FDR-adjusted p-values <0.05) and using ANOVA test for the human LN kidney dataset (with fold change difference >|1.5| and FDR-adjusted p-values <0.05. Venn Diagram shows the number of differentially expressed genes common between kidneys from pristane-treated SNF1 mice compared to age matched controls, kidneys from Adv-IFNα BWF1 mice compared to age matched controls, and kidney glomeruli tissues from LN patients compared to normal human kidney glomeruli tissues. (B) Gene Set Enrichment Analysis (GSEA) was used to detect whether the upregulated (UP) and downregulated (DN) genes in the human LN were enriched in the top upregulated and top downregulated genes in the kidneys from pristane-treated SNF1 mice respectively. (C) Z-score heatmap showing expression profiles of genes in kidneys from SNF1 mice with and without pristane treatment. All genes shown were differentially expression with a fold change greater than 2 between kidneys from SNF1 at 14 week post-pristane injection vs control (FDR-adjusted p-value<0.05).

We compared the kidney transcript profiles from the accelerated mouse models to a publicly available microarray dataset of glomeruli from LN patients and normal subjects [25]. Differentially expressed genes in the mouse models compared to the human dataset were defined for genes for which there were probes present on both human and mouse platforms Fig 4A and Sup S1 File). Gene Set Enrichment Analysis confirmed that the upregulated and downregulated human LN signatures were strongly enriched in the pristane-SNF1 kidney strongest upregulated and downregulated genesets respectively (FDR adjusted p-value<0.01 for gene set enrichment analysis) (Fig 4B) [26,27]. Biological processes that are upregulated in human LN, such as IFN response, complement, Fc gamma receptors, immune recruitment, innate immune pattern recognition, antibody response and fibrosis, were upregulated in kidneys of both the pristane-SNF1 model and ADV-IFNα BWF1 models (Fig 4C and S3 Fig).

To identify genes that may be specifically modified in one model over the other, we selected genes that showed at least 2 or 3 fold difference in their response to pristane or ADV-IFNα, depending on whether they passed statistical significance in only one mouse model or both models. There were 127 genes and 55 genes that appeared more specific to the pristane-SNF1 model or the Adv-IFNα BWF1 model, respectively. Only a subset of these genes had probes also present in the human microarray platform with 4 genes differentially expressed in human LN and more specific to the Adv-IFNα BWF1 model (*CEBPD*, *CYR61*, *DDX60*, *IGJ*) and 14 genes differentially expressed in human LN and more specific to the pristane-SNF1 model (S1 File). Several of the pristane-SNF1 specific genes were genes involved in innate immunity, such as *Sting*, *Aim2* and inflammatory mediators such as *S100a8/S110a9* and *Saa1/Saa3*. *AIM2 and S100A9* were upregulated in subsets of patient samples in the human dataset but did not pass statistical significance (there was no probe for STING on the human microarray platform) (Sup S1 File). In addition and consistent with the pathology assessment, fibrosis genes *Col1a2*, *Tnc*, *Acta2*, *Fbn2*, *Timp1*, *Kim1* were specifically upregulated in the pristane-SNF1 model (S1 File). *TNC*, *TIMP1*, *ACTA2*, *COL1A2* were also upregulated in the human LN dataset (no probe for KIM1 on the human microarray platform) (S1 File and detailed in later section).

Given that transcriptional signatures from peripheral blood of SLE patients have been extensively characterized [28–30], we also aimed to determine the gene expression profiles of the peripheral blood from pristane-SNF1 mice, from matched control mice as well as from 36-week old SNF1 mice with spontaneous disease. We detected 1410 differentially expressed genes in the peripheral blood of SNF1 mice at 14 weeks after pristane injection (> 2-fold expression change compared to age-matched controls, FDR adjusted p<0.05) (S1 File). Similar to the human SLE blood signatures, interferon responsive genes, plasma cell genes and neutrophil signatures were upregulated, while the T-cell signature and genes associated with protein synthesis were downregulated in the blood of pristane-treated SNF1 mice (S4 Fig). In contrast, 36-week old SNF1 mice showing spontaneous disease did not show the same level of transcriptional alterations in the peripheral blood (S4 Fig).

### Pristane treatment in SNF1 mice induces upregulation of nucleic acid sensors Aim2 and Sting

**Fig 5:**
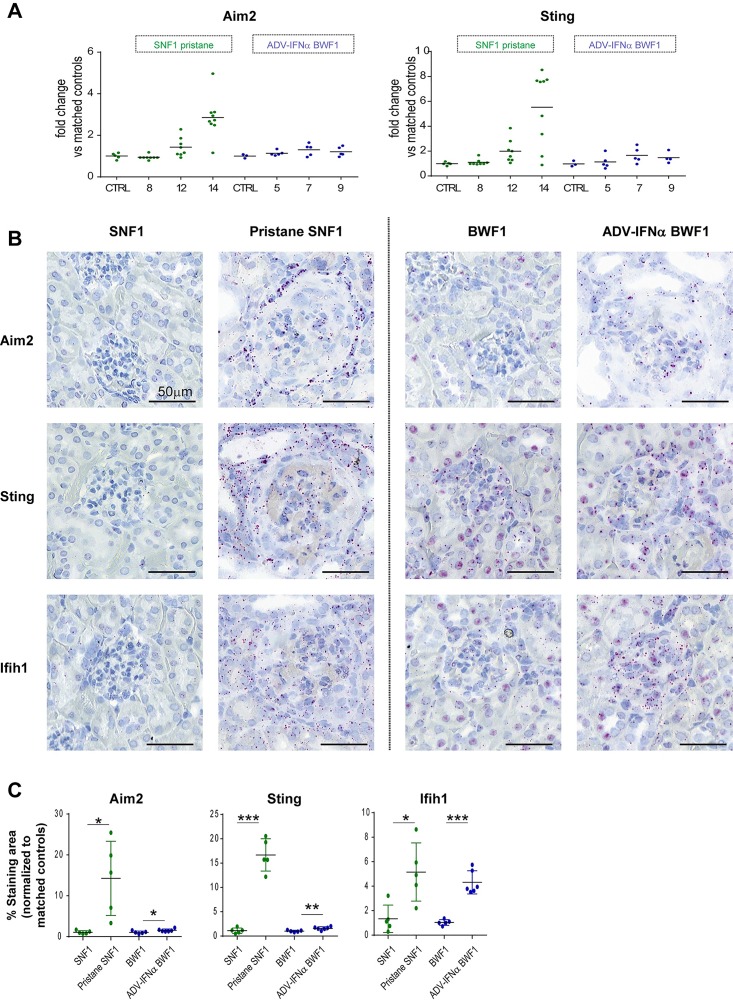
Innate immune sensors *Aim2* and *Sting* are upregulated in pristane-SNF1 model. (A) Gene expression changes of *Aim2* and *Sting* in the kidneys of pristane-treated SNF1 mice and Adv-IFNα BWF1 mice. Data are shown as expression fold change compared to matched control untreated mice. (B) Aim2 and Sting transcripts were detected by RNAscope in kidney tissue sections from SNF1 with or without pristane treatment (week 14) and BWF1 mice with or without Adv-IFNα injection (week 9). Diseased animals were matched for proteinuria levels. (C) Results from image quantification from RNAscope hybridization described in (B) with Aim2, Sting and Ifhi1 RNAscope staining area normalized to the mean of the staining area from the control groups. Each dot represents an animal. (* p-value<0.05, **p-value<0.01, unpaired two-tailed Student’s t-test).

The results from microarray analysis of the kidney tissues from pristane-SNF1 model and Adv-IFNα BWF1 model suggested that *Aim2* and *Sting* RNA expression was more upregulated in the pristane-SNF1 model (Fig 5A). Due to the lack of reliable reagents for Aim2 and Sting immunohistochemistry in formalin fixed tissues, we used RNAscope in-situ hybridization to detect *Aim2* and *Sting* transcripts. The baseline expression of *Aim2 and Sting* was higher in the untreated BWF1 kidney tissues than in the untreated SNF1 mice (Fig 5B). However, *Aim2* and *Sting1* expression did not increase strongly in the diseased Adv-IFNα BWF1 kidney tissues (less than 2 fold increase vs. matched controls) compared to 13–16 fold increase in the pristane-SNF1 kidneys vs matched controls (Fig 5C). As an internal control for RNA integrity and disease status, we confirmed that the interferon responsive gene *Ifih1* was upregulated in both models (Fig 5B and Fig 5C).

Thus, pristane treatment is able to induce a strong upregulation of the expression of the nucleic acid sensors *Aim2* and *Sting* in SNF1 mice, which is not observed in the Adv-IFNα BWF1 model.

### Pristane treatment in SNF1 mice induces fibrosis resembling human LN

**Fig 6:**
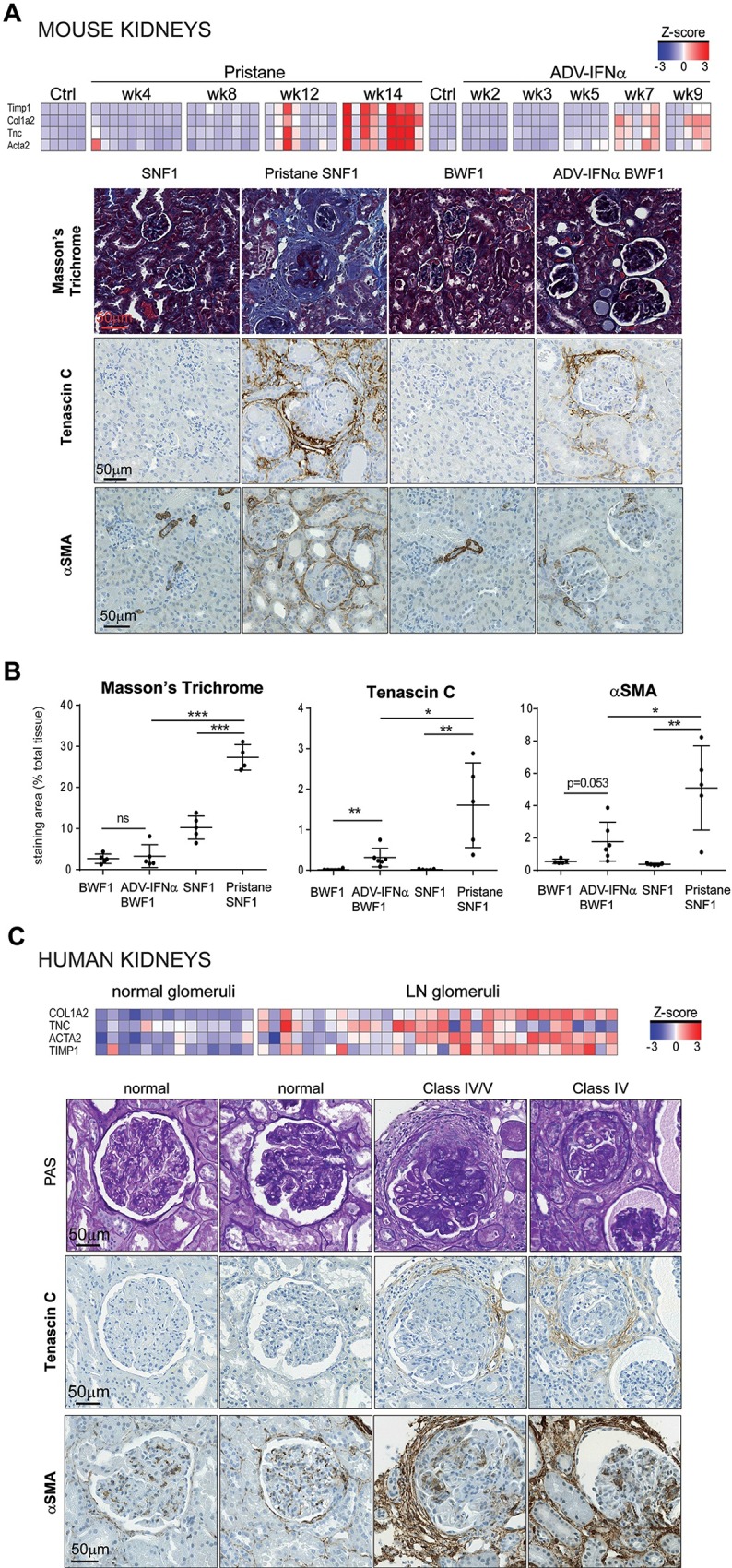
Pristane treatment of SNF1 mice induces human lupus nephritis-like fibrosis. (A) Gene expression levels of *Tnc*, *Timp1*, *Col1a2*, *Acta2* in the kidneys of pristane-treated SNF1 mice and Adv-IFNα BWF1 mice (Heatmap shows expression levels in Z-scores). Masson’s Trichrome stain and Tenascin C and alpha smooth muscle actin (ASMA) immunohistochemistry (IHC) were performed on kidney tissue sections from SNF1 with or without pristane treatment (week 14) and BWF1 mice with or without Adv-IFNα injection (week 9). Diseased animals were matched for proteinuria levels. (B) Staining or immunopositive area was quantified in percentage of total tissue. Each dot represents an animal. (* p-value<0.05, **p-value<0.01, unpaired two-tailed Student’s t-test). (C) *TNC*, *TIMP1*, *COL1A2*, *ACTA2* gene expression levels in normal kidney glomeruli tissues and LN kidney glomeruli tissues from public human microarray dataset. (Heatmap shows expression levels in Z-scores). PAS and tenascin C and ASMA IHC were performed on biopsies from LN patients.

Transcripts encoding fibrosis markers such as Tnc, Kim1, Col1a2, Acta2, Timp1 had a greater increase in kidneys of pristane-SNF1 model compared to the ADV-IFNα BWF1 model (Fig 6A). Microarray data from the human LN-glomeruli tissues showed that the increase of *TNC*, *TIMP1*, *ACTA2*, *COL1A2* expression also occurs in human disease (Fig 6C). There was no probe for *KIM1* on the human microarray platform.

Using Masson’s Trichrome staining and immunohistochemistry, we confirmed that tissue fibrosis was a prominent feature of pristane-SNF1 mice. There was more extracellular matrix deposition (Masson’s Trichrome and tenascin C) as well as more alpha smooth muscle actin (ASMA) immunoreactivity (fibroblasts, pericytes) in the kidneys of pristane-SNF1 mice relative to the ADV-IFNα BWF1 model (Fig 6A and 6B). In both the pristane-SNF1 model and the human disease tissues, tenascin C and alpha smooth muscle actin were detected within the interstitial fibrous connective tissue between tubules and surrounding injured glomeruli (Fig 6A and 6C). Interestingly, in our limited panel, (5 patients), Tenascin C was only observed in scattered interstitial foci in patients with Class II and Class III diagnoses (not shown), whereas in the 2 patients with Class IV and IV/V diagnoses, it was prominent in the periglomerular and inter-tubular interstitium (Fig 6C). Thus, the pristane-SNF1 model showed a robust fibrosis component that is also observed in the human disease, perhaps especially in Class IV/V diagnoses.

## Discussion

This study describes a novel accelerated model of lupus that overlays pristane, a known inducer of autoimmunity, onto the genetic background of SNF1 lupus prone mice. While pristane treatment in BWF1 mice has been previously shown to accelerate disease, we prioritized the characterization of pristane treatment in the SNF1 background as it led to faster disease acceleration. The pristane-treated SNF1 mice develop proteinuria 5 months earlier than untreated animals and 6 weeks earlier than pristane-treated BWF1 mice. The pristane-SNF1 model was compared with another well-established and widely used accelerated model, Adv-IFNα BWF1. While pristane-SNF1 model recapitulated many features of Adv-IFNα BWF1, there were specific features of the pristane-SNF1 model that better represented human disease, such an upregulation of the cytosolic nucleic acid sensor pathway, tubulointerstitial inflammation and robust fibrosis.

**Fig 7:**
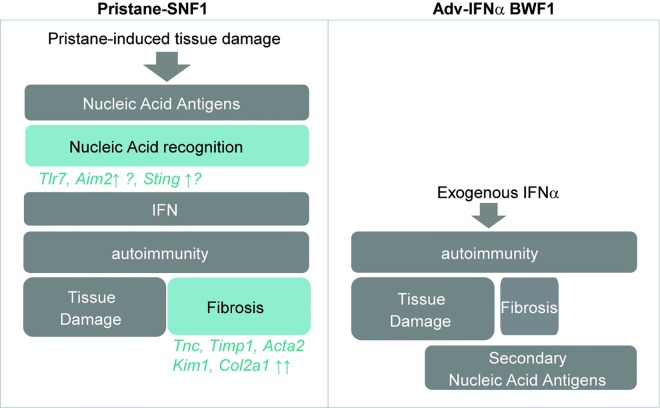
Summary of the potential pathogenesis mechanisms involved in pristane-SNF1 and Adv-IFNα BWF1 disease models.

Pristane treatment in SNF1 mice led rapid and dramatic increase in total Ig and ANA levels. Pristane has been shown to induce apoptosis in treated mice [31]. Increased apoptosis mediated by pristane treatment in conjunction with defective clearance of apoptotic cells documented in SNF1 mice likely amplify the source of autoantigens, resulting in loss of tolerance, increase in nephritogenic autoantibodies and disease acceleration [32]. One of the distinctive features of the pristane-SNF1 model compared to the Adv-IFNα BWF1 model was the increased expression of *Aim2* and *Sting* in diseased kidneys. Both AIM2 and STING are known to contribute to the downstream activation of immune signal pathways in response to apoptotic DNA. In pristane SNF1, it is possible that large amounts of apoptotic debris activates nucleic acid sensing pathways that can in turn trigger downstream IFN-I production and autoimmunity. In contrast, the Adv-IFNα BWF1 model may not require initial activation of the nucleic acid sensing pathways for disease acceleration as the induction of autoimmunity is triggered by exogenous IFN-I rather than endogenous IFN-I production (see model in Fig 7). The role of interferon in pristane-induced autoimmunity has been previously demonstrated, where pristane-treated *Ifnra2*-/- mice showed reduced total IgG levels compared to pristane treatment in C57BL/6 wt mice [33]. In addition, Tlr7, another nucleic acid sensors located in endosomes, has also been shown to contribute to IgG and ANA production as well as kidney IC deposition and glomerulonephritis in pristane-induced disease in C57BL/6 wt mice [13,34]. Further studies may be needed to better understand the relative contribution of cytosolic and endosomal nucleic acid sensing in the pristane-SNF1 mice. Overall, with accumulating evidence of a potential role of the cytosolic nucleic acid sensing pathway in the development of human lupus [35], the pristane-SNF1 model might be a particularly appropriate model to study the role of these cytosolic nucleic acid sensors in disease and the potential effect of modifiers of this pathway on disease progression. The pathogenic mechanisms in pristane-SNF1 mice are consistent with human lupus given that genome-wide association studies (GWAS) have highlighted genetic variants associated with higher risk for SLE that are linked to apoptotic clearance, innate immune sensing and the IFN pathway [36,37].

A striking specific feature of the pristane-SNF1 model is the prominent tubulointerstitial inflammation and fibrosis underscored by morphologic observations and kidney gene expression profiles. Detection of the fibrosis markers Masson’s Trichrome stain, tenascin C, a hallmark extracellular matrix protein and ASMA positive cells confirmed that the pristane-SNF1 model develops a strong fibrotic response than the Adv-IFNα BWF1 model. ASMA staining also highlighted a much stronger presence of perhaps myofibroblasts in the pristane-SNF1 model compared to the Adv-IFNα BWF1 model. Interstitial inflammation and fibrosis in the pristane-SNF1 model are likely connected as fibrosis is thought to be an aberrant tissue repair response that can be triggered by tissue injury upon chronic renal inflammation [38,39]. Both interstitial inflammation and fibrosis are associated with poor disease outcome in human LN [21–24]. Here we demonstrated that tubulointerstitial fibrosis in both human LN and in pristane-SNF1 was accompanied by upregulated expression of patterns of genes consistent with fibrosis. Immunosuppressive treatments in autoimmune LN NZBWF1/J mice model and in human LN were shown to reverse kidney fibrotic lesions to some extent [40,41], which further reflects the interdependency of inflammation and fibrosis in nephritic disease. To establish whether the pristane-SNF1 kidney fibrotic lesions are reminiscent of the human disease, we confirmed that the expression of the fibrosis genes observed in the pristane-SNF1 model were also upregulated in a human LN microarray dataset and that the immunostaining of tenascin C and alpha smooth muscle actin proteins showed similar localization in renal biopsies from LN patients. Several studies have shown that renal interstitial fibrosis or a composite chronicity index that includes interstitial fibrosis as a component are both predictive of renal failure outcome and survival [22–24,42]. In addition, the presence of residual fibrosis in repeated biopsies has been also associated with a poor clinical outcome [41]. Together, this suggests that an efficacious LN therapy would require effective modulation of both kidney tubulointerstitial inflammation and fibrosis.

Since the pristane-SNF1 model exhibits robust accelerated interstitial inflammation coupled with fibrosis, it is an attractive model for studying the role of fibrosis in the development of autoimmune-induced nephritis. Kidney fibrosis in 48 week old SNF1 mice and could be inhibited by anti-CD40L, supporting the hypothesis that fibrosis in this model is linked to sustained auto-immune injury [43,44]. Kidney transcript profiling of other longer spontaneous models such as 36 week old NZB/W, 30 week old NZM2410 and 18–21 week NZW/BSB mice also showed a kidney fibrosis response, but the fold increase in expression was 2–7 fold higher in the pristane-SNF1 model for *Tnc* and *Timp1* than has been reported in the spontaneous models [45]. To our knowledge, there has been no report of a well-characterized fibrotic response in accelerated mouse models of lupus nephritis. The unilateral ureter obstructive model (UUO), a 6–10 day model, has been the most widely used model to examine mechanisms of tubulointerstitial fibrosis *in vivo*. While this model offers a rapid, robust, and reproducible phenotype it is not a relevant model of lupus and does not lead to proteinuria or significant glomerulopathy [46]. The pristane-SNF1 accelerated model therefore provides a unique opportunity to explore the potential inflammation/fibrosis modifiers in the context of lupus nephritis.

In summary, pristane-SNF1 is a new model of LN characterized by a rapid onset and progression to renal disease. This model displays many human disease features such as blood SLE transcriptional signatures, kidney interstitial inflammation and fibrosis with a likely involvement of apoptotic clearance and nucleic acid sensing pathways. Since this model responds to therapeutics that may be of benefit in human SLE, this system provides a valuable experimental tool to further understand the mechanisms contributing to both rodent and human LN.

## Supporting Information

S1 Fig Detection of positive staining tissue area using image analysis. Top panel: Left panels show representative pictures of *Ifih1* RNAscope staining. Right panels show identification of RNAscope positive area (red) using Image J RNAscope quantification method. Bottom panel: Left panel shows Masson’s Trichrome staining of kidney tissue from SNF1-pristane animal, right panel shows identification of positive staining area.
(TIF)

S2 Fig Onset of proteinuria in pristane-treated BWF1 and SNF1 mice. Progression of proteinuria pristane-treated BWF1 mice and pristane-treated SNF1 mice. Each symbol indicates an individual mouse.
(TIF)

S3 Fig Kidney transcriptional response from Adv-IFNα BWF1 model. Z-score heatmap showing gene expression profiles in kidneys from BWF1 mice with and without Adv-IFNα injection.
(TIF)

S4 Fig Blood expression profiles of genes from human SLE gene signatures in the blood of pristane-treated SNF1 mice. (A) Z-score heatmap showing gene expression profiles in the blood from SNF1 mice with and without pristane treatment. Genes were selected from the gene modules reported to be altered in human SLE patients. (B) Expression changes of genes from modules altered in human SLE in during the disease progression in the blood pristane-treated mice, and in 36-week old SNF1 mice with spontaneous disease. Data are shown in expression fold change compared to matched control untreated mice.
(TIF)

S1 File Microarray data results. Table A contains the results from microarray data analysis comparing the gene expression profiles from kidneys of pristane-SNF1 mice (14 weeks after treatment) vs untreated SNF1 mice and from kidneys of Adv-IFNα BWF1 mice (9 weeks after treatment) vs untreated BWF1 mice. Table B contains the results from microarray data analysis comparing gene expression profiles from whole blood of pristane-SNF1 mice (14 weeks after treatment) vs untreated SNF1 mice. Table C contains the analysis results from publicly available microarray data comparing glomeruli tissues from LN patients vs non-diseased subjects. Table D contains the mouse genes for which human ortholog genes were found in both mouse and human microarray platforms. Table E contains the list of genes differentially expressed in the disease kidneys that are either more specific of the pristane-SNF1 model or of the Adv-IFNα BWF1 model.
(XLSX)

## Abbreviations

SLE: Systemic lupus erythematosus
LN: lupus nephritis
Adv: adenovirus
IC: Immune complexes
rIFNα: recombinant IFNα
Adv-IFNα BWF1: IFNα treated NZB X NZW F1 mice
SNF1 mice: (SWR X NZB F1) mice
ASMA: alpha smooth muscle actin
