# Supplementary figures and images for: Pristane-Accelerated Autoimmune Disease in (SWR X NZB) F1 Mice Leads to Prominent Tubulointerstitial Inflammation and Human Lupus Nephritis-Like Fibrosis
Source: PLoS One. 2016 Oct 19;11(10):e0164423. doi: 10.1371/journal.pone.0164423 (PMC5070861; doi:10.1371/journal.pone.0164423)

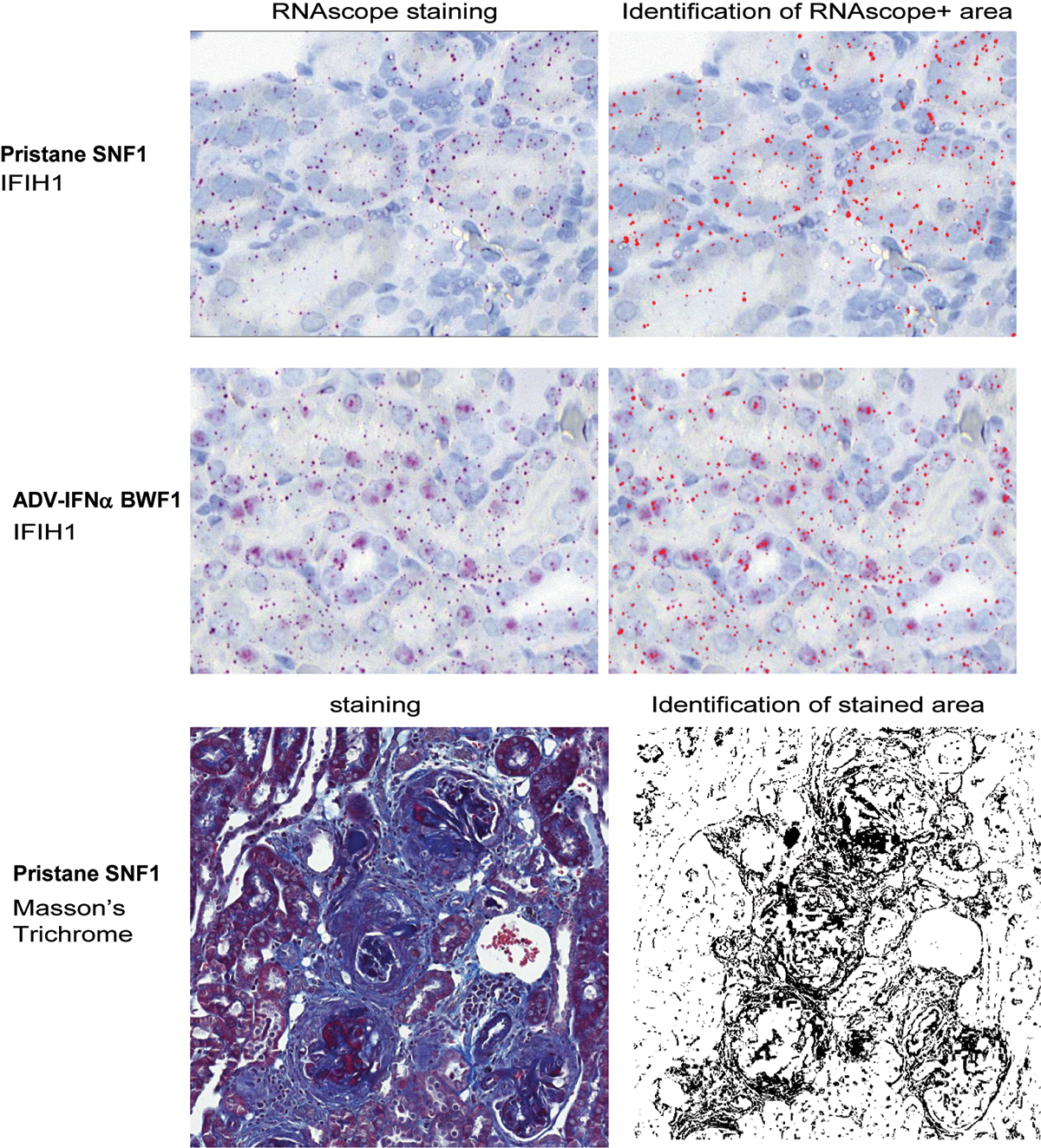

Supplement: S1 Fig — Top panel: Left panels show representative pictures of Ifih1 RNAscope staining. Right panels show identification of RNAscope positive area (red) using Image J RNAscope quantification method. Bottom panel: Left panel shows Masson’s Trichrome staining of kidney tissue from SNF1-pristane animal, right panel shows identification of positive staining area. (TIF) [file pone.0164423.s001.tif]

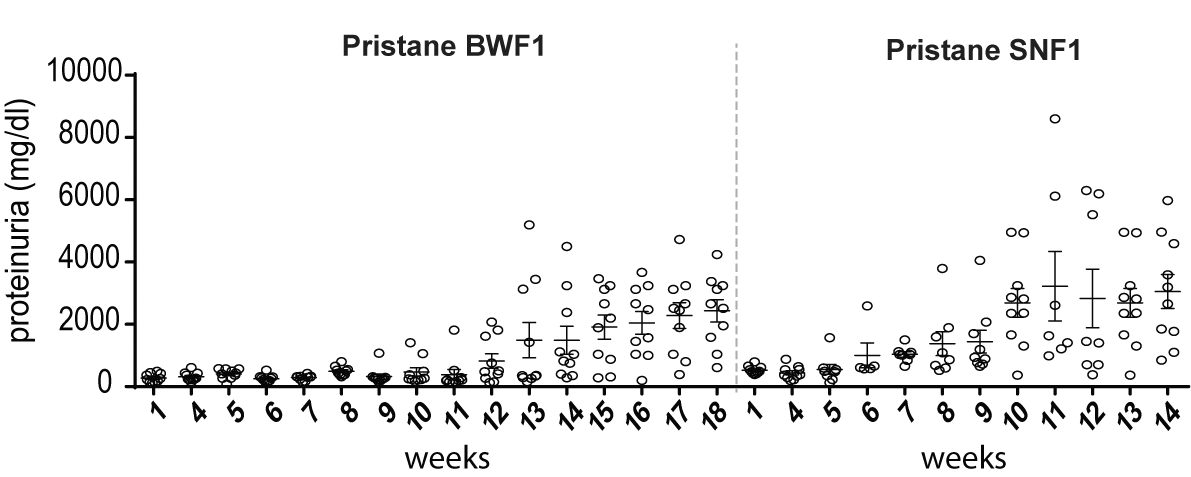

Supplement: S2 Fig — Progression of proteinuria pristane-treated BWF1 mice and pristane-treated SNF1 mice. Each symbol indicates an individual mouse. (TIF) [file pone.0164423.s002.tif]

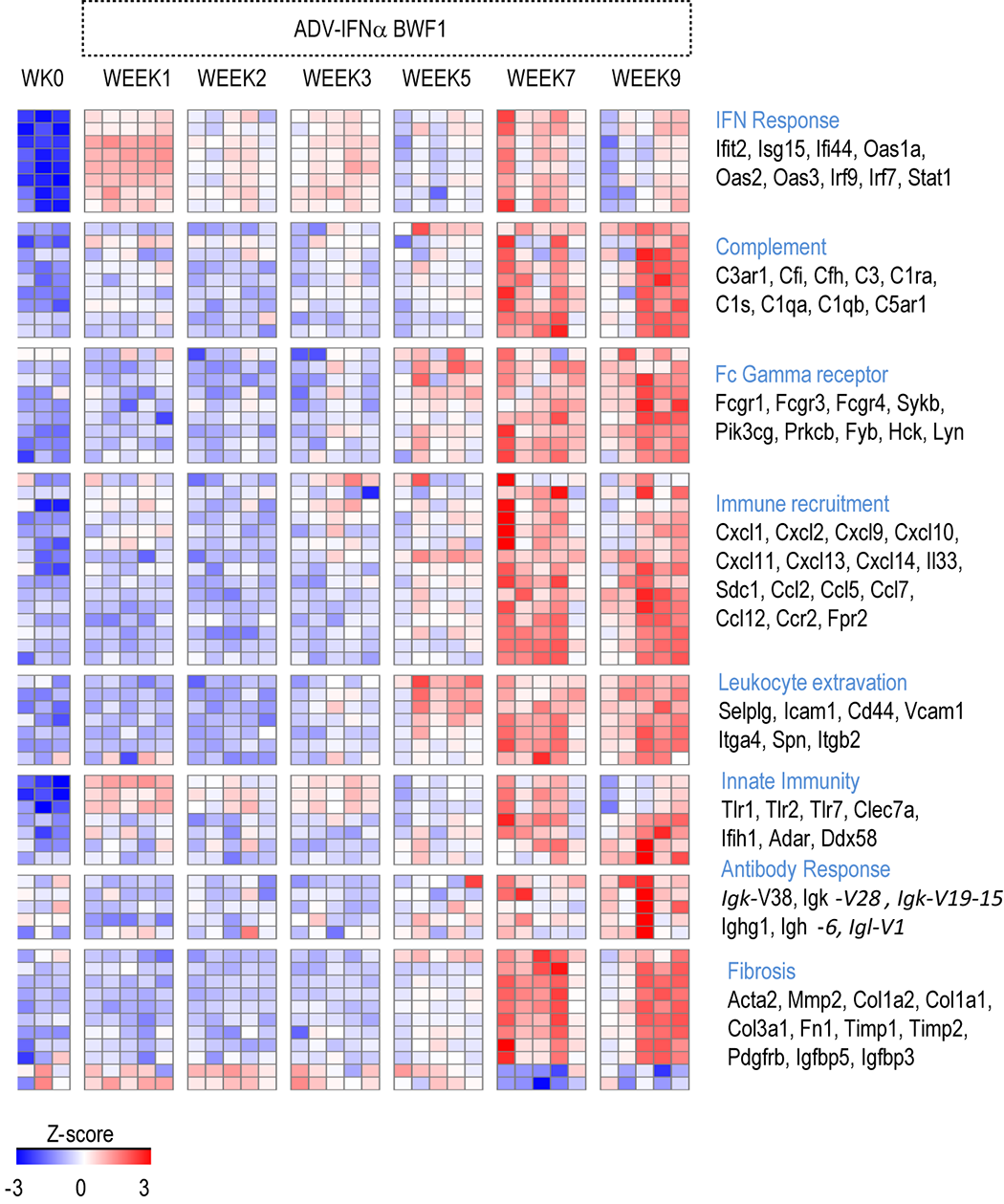

Supplement: S3 Fig — Z-score heatmap showing gene expression profiles in kidneys from BWF1 mice with and without Adv-IFNα injection. (TIF) [file pone.0164423.s003.tif]

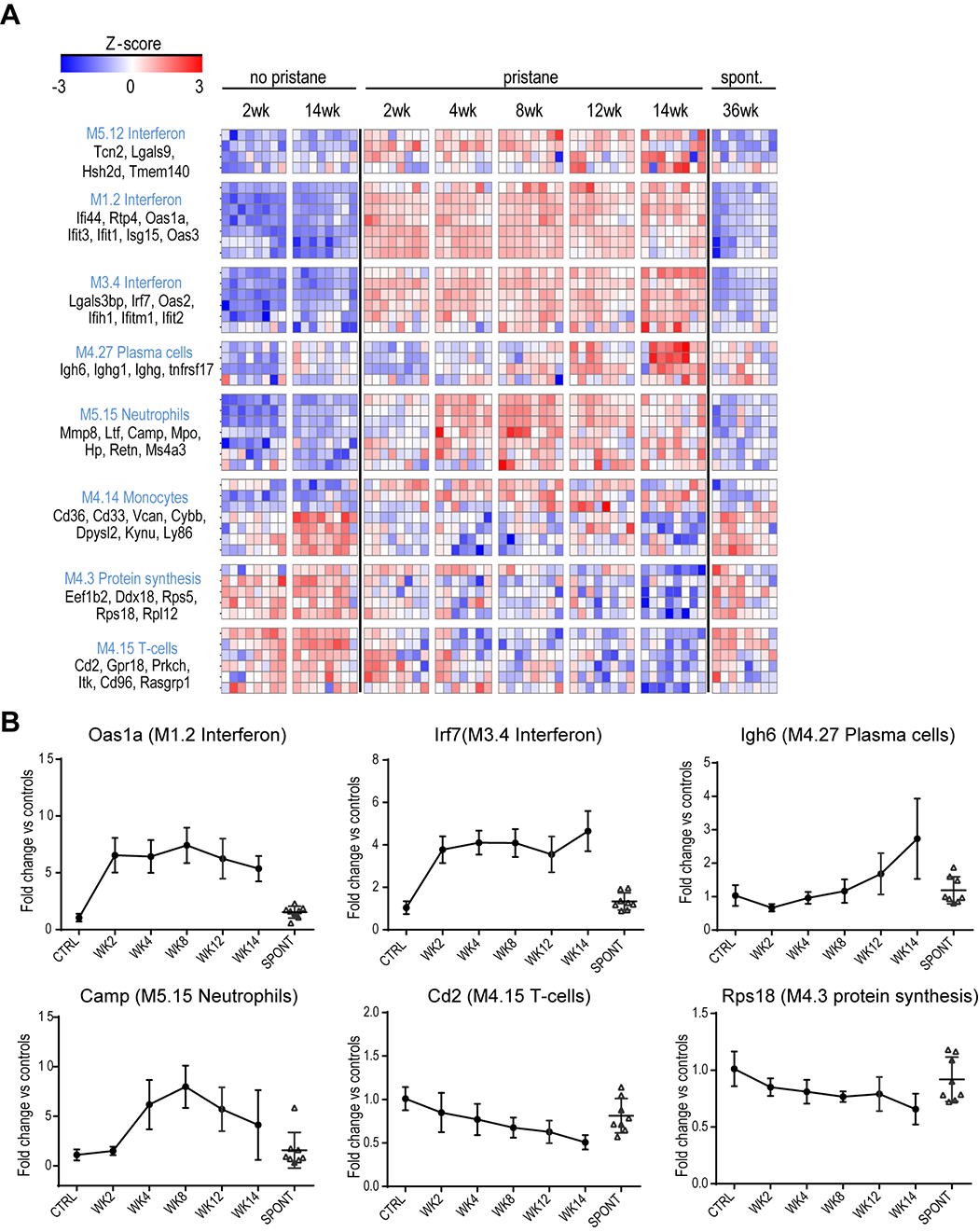

Supplement: S4 Fig — (A) Z-score heatmap showing gene expression profiles in the blood from SNF1 mice with and without pristane treatment. Genes were selected from the gene modules reported to be altered in human SLE patients. (B) Expression changes of genes from modules altered in human SLE in during the disease progression in the blood pristane-treated mice, and in 36-week old SNF1 mice with spontaneous disease. Data are shown in expression fold change compared to matched control untreated mice. (TIF) [file pone.0164423.s004.tif]
